# THE INTERPLAY AMONG IMMUNITY, INFLAMMATION, AND FRIED FRAILTY ON ALZHEIMER’S DISEASE

**DOI:** 10.1093/geroni/igae098.0731

**Published:** 2024-12-31

**Authors:** Jiachen Chen, Margaret Doyle, Yumeng Cao, Ahmed Ragab, Joanne Murabito, Kathryn Lunetta

**Affiliations:** Boston University School of Public Health, Boston, Massachusetts, United States; University of Vermont Larner College of Medicine, Colchester, Vermont, United States; Boston University, Boston, Massachusetts, United States; Boston University, Boston, Massachusetts, United States; Chobanian and Avedisian School of Medicine, Boston University, Boston, Massachusetts, United States; Boston University, Boston, Massachusetts, United States

## Abstract

Unraveling shared mechanisms for both frailty and Alzheimer’s Disease (AD) including immunity and inflammation is crucial for understanding the link between these two complex multifactorial conditions. This study aimed to characterize the interplay among circulating immune cells, inflammatory biomarkers, and frailty phenotypes on AD onset in the Framingham Heart Study Offspring cohort. A sample of 519 participants (52% female, mean age 66 years), had extensive profiling of immune cells from peripheral blood mononuclear cells and inflammatory protein biomarkers (OLINK Proteomics) at Exam 7 and were dementia-free at the time of Fried frailty measurement (Exam 8). Data from 146 immune cells, 68 proteins, and 5 frailty phenotype components were integrated using sparse multiple Canonical Correlation Analysis (SmCCA) adjusted for relevant covariates. SmCCA seeks linear combinations of variables maximizing inter-correlated canonical scores for all three datasets, yielding signatures of immunity, inflammation, and frailty for each participant that screened variables contributing to the establishment of signatures and inter-datasets pathways. Full follow-up for up to 13.8 years (median 12.1 years) identified 24 incident AD cases. Cox proportional hazard models, accounting for covariates including age, sex, education, and APOE genotype, were employed to assess signatures as risk factors for AD incidence. The inflammation and immunity signatures were correlated (r2=0.32, p-value< 0.001). Both immunity and frailty signatures were significantly associated with AD incidence (immunity: HR=1.1, p-value=0.03; frailty: HR=3.14, p-value=0.02). This study disentangles potential pathways among immune cells and inflammatory biomarkers that contributed to associations with frailty and AD. We will extend to larger studies for replication.

